# Effect of an Arm-Span-Adjusted Hand Position on Closed Kinetic Chain Upper Extremity Stability Test Performance and Muscle-Related Body Composition Associations

**DOI:** 10.3390/muscles5020037

**Published:** 2026-05-08

**Authors:** Filippos Christodoulou, Petros Maniatis, Andreas Erotocritou, Stelios Hadjisavvas, Michalis A. Efstathiou, Irene-Chrysovalanto Themistocleous, Manos Stefanakis

**Affiliations:** Department of Health Sciences, Physiotherapy Program, University of Nicosia, 46 Makedonitissas Avenue, 1700 Nicosia, Cyprus; filipposchristodoulou@yahoo.gr (F.C.); petros_maniatis@hotmail.com (P.M.); antreaser@gmail.com (A.E.); efstathiou.m@unic.ac.cy (M.A.E.); themistocleous.i@unic.ac.cy (I.-C.T.); stefanakis.m@unic.ac.cy (M.S.)

**Keywords:** closed kinetic chain upper extremity stability test, CKCUEST, upper extremity, functional performance, hand position, anthropometry, body composition

## Abstract

Background: The Closed Kinetic Chain Upper Extremity Stability Test (CKCUEST) is used to assess upper-extremity performance in a closed kinetic chain position. The standard hand placement of 36 inches may favor individuals with larger body dimensions. Methods: Sixty-five healthy adults (44 males, 21 females; 18–33 years) performed the CKCUEST under two conditions: the standard position and a modified position with hand distance set at 50% of arm span. The mean number of touches, standard score, and power score were calculated for each condition. Reliability and the effects of sex and body composition were also examined. Results: Performance was significantly better in the modified position for mean touches (24.4 ± 4.47 vs. 23.0 ± 4.62, *p* = 0.001), standard score (0.4 ± 0.07 vs. 0.3 ± 0.06, *p* = 0.001), and power (81.1 ± 18.29 vs. 77.1 ± 22.00, *p* = 0.001). Both conditions showed excellent reliability (ICC = 0.944–0.946). Females performed significantly fewer touches than males in the standard position (*p* = 0.001), whereas this difference was not significant in the modified position. Several anthropometric and body composition variables significantly predicted performance. Conclusions: An arm-span-adjusted hand position improves CKCUEST performance and may provide a fairer assessment across individuals with different body dimensions.

## 1. Introduction

Functional tests are commonly used in the assessment of athletes and individuals with musculoskeletal disorders because they provide an objective evaluation of functional performance. They are relatively simple to administer and interpret in both field and clinical settings, and they can detect changes over time, thereby informing rehabilitation and return-to-sport decisions [1,2,3].

The evaluation of upper-extremity musculoskeletal function commonly includes the assessment of movement-related variables, and the results of clinical and functional tests [4]. Among the available upper-extremity performance tests, the Closed Kinetic Chain Upper Extremity Stability Test (CKCUEST) is widely used to assess upper-extremity stability and performance during closed kinetic chain tasks [5,6,7]. The test is practical, requires minimal equipment, and can be applied in both sports and clinical settings [6,8].

The CKCUEST has demonstrated strong test–retest reliability across different populations [6,8,9]. Its validity is also supported by significant associations with grip strength, isokinetic shoulder strength, and other upper-extremity functional tests, such as the Upper Quarter Y Balance Test and the Seated Medicine Ball Throw test [6,10,11]. In addition, the test has shown discriminative ability between physically active and sedentary individuals, as well as between asymptomatic individuals and those with shoulder symptoms, and has been studied in relation to injury risk and return-to-sport decision-making [8,12,13,14].

Despite its wide use, the standard CKCUEST protocol requires a fixed hand distance of 36 inches (91.44 cm), which may favor individuals with larger body dimensions and place shorter individuals at a mechanical disadvantage. Few studies have examined the effect of hand distance on test performance. Tucci et al. [4] reported no significant differences in kinetic and kinematic variables across different hand distances, although their study focused mainly on biomechanical rather than performance outcomes. Hollstadt et al. [9] reported acceptable reliability for a modified version performed with the hands directly below the shoulders, but without direct comparison with the traditional position in a large sample. More recently, Callaway et al. [15] compared different starting positions and found that hand placement influenced performance, with the 50%-of-height position showing favorable reliability characteristics.

Although the CKCUEST has been previously investigated in terms of reliability, validity, clinical utility, and modified starting positions, the present study does not introduce a completely new upper-extremity performance test. Rather, it extends previous CKCUEST research by directly comparing the traditional fixed 36-inch hand distance with an individualized hand distance based on 50% of arm span. This approach was designed to examine whether scaling hand placement to body dimensions can reduce the potential anthropometric bias of the standard protocol. In addition, by examining sex differences and body-composition associations, the study further explores methodological factors that may influence CKCUEST interpretation.

Therefore, as an extension of previous CKCUEST research, the primary aim of the present study was to examine the effect of hand position on CKCUEST performance by comparing the standard 36-inch position with a modified position in which hand distance was adjusted to 50% of arm span. The secondary aims were to investigate the effects of sex and body composition on test performance. It was hypothesized that the arm-span-adjusted position would result in better performance and reduce the influence of anthropometric differences on test outcomes.

### Research Questions and Hypotheses

Based on the existing literature and the identified gaps, the following research questions were formulated:

1. Does an arm-span-adjusted hand position affect CKCUEST performance compared with the standard 36-inch position?
2. Does the modified hand position reduce sex-related differences in CKCUEST performance?
3. What is the relationship between anthropometric/body composition variables and CKCUEST performance in each testing condition?

Accordingly, the following hypotheses were proposed:

**H1:**  *CKCUEST performance (mean touches, standard score, and power) will be significantly higher in the arm-span-adjusted condition compared with the standard condition*.

**H2:**  *Protocol-dependent group differences in CKCUEST performance between male and female participants will be reduced in the arm-span-adjusted condition compared with the standard condition*.

**H3:**  *Anthropometric and body composition variables will be significantly associated with CKCUEST performance, with weaker associations observed in the arm-span-adjusted condition*.

## 2. Methods

### 2.1. Study Design and Participants

This cross-sectional study included 65 healthy volunteers (44 males and 21 females) aged 18–33 years. Participants were recruited through announcements distributed in gyms, fitness facilities, and sports centers in the districts of Nicosia and Larnaca, Cyprus. All participants were physically active and exercised at least twice per week. The study was conducted in accordance with the Declaration of Helsinki and was approved by the Cyprus National Bioethics Committee (ΕΕΒΚ/ΕΠ/2025/31). Written informed consent was obtained from all participants prior to participation.

### 2.2. Inclusion and Exclusion Criteria

Eligible participants were healthy adults aged 18–33 years who were able to understand and follow verbal instructions in either Greek or English and who engaged in physical exercise at least twice weekly. Participants were excluded if they reported any current or recent upper- or lower-limb musculoskeletal injury, cervical radiculopathy, low back pain, nerve entrapment syndrome, recent upper-extremity or core injury or surgery, shoulder instability or dislocation, or any vascular or cardiac condition that could affect safe participation in the testing procedure.

### 2.3. Testing Procedure

All assessments were conducted in the morning between 8:00 a.m. and 12:00 p.m. at the University of Nicosia. Participants were instructed to avoid food, alcohol, and caffeine after midnight before testing and to refrain from strenuous physical activity on the preceding day. Testing was performed with light clothing and without shoes or socks. Height and arm span were measured using a portable stadiometer (Seca, Hamburg, Germany). Body composition was assessed using a calibrated electronic scale (Omron BF511, Omron Healthcare Co., Ltd., Kyoto, Japan), which provided measurements of body mass (kg), lean body mass (kg), body fat mass (kg), body fat percentage, and visceral fat level. Body mass index (BMI) was calculated as body mass in kilograms divided by height in meters squared (kg/m^2^).

**Figure 1:**
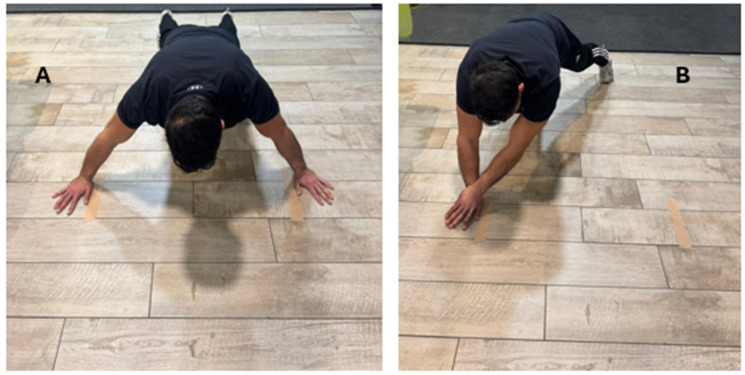
Body position of participants before and during the test. (**A**) Initial body position. (**B**) Body position during the test.

Before testing, participants completed a standardized 10-min warm-up consisting of shoulder, trunk, and upper-extremity mobility exercises. The CKCUEST was then performed under two conditions: (1) the standard position, with the hands placed 36 inches (91.44 cm) apart, and (2) a modified position, with hand distance set at 50% of each participant’s arm span. Arm span was measured as the distance between the tips of the middle fingers with the shoulders abducted to 90°. Both hand positions were marked on the floor with tape. The initial and testing body positions are illustrated in Figure 1.

Participants assumed the test position with the hands placed just outside the marked lines. Male participants performed the test in the standard push-up position, whereas female participants performed it in the modified push-up position, according to the original test administration procedure used in previous studies. Because male and female participants performed the CKCUEST using different body positions, sex-based comparisons were interpreted as protocol-dependent comparisons rather than direct physiological comparisons between sexes. All participants were instructed to maintain a straight trunk and to alternately reach across with one hand to touch the supporting hand as quickly as possible for 15 s. Two examiners administered the test: one counted the number of touches and the other controlled the time and provided standardized verbal commands.

Before the experimental trials, all participants performed two familiarization trials of 15 s each, one at approximately 50% of maximal speed and one at maximal speed, separated by 1 min of rest. After a 2-min rest period, the testing phase began. The order of the two hand distance conditions was randomized using a coin toss. Each condition was performed three times, with 45 s of rest between trials and 5 min of rest between conditions.

### 2.4. Outcome Measures

For each testing condition, the number of touches completed in each of the three 15-s trials was recorded. The mean number of touches across the three trials was used for analysis. The standard score was calculated by dividing the mean number of touches by participant height. Power was calculated by multiplying 68% of body mass by the mean number of touches and dividing the result by 15, according to the original CKCUEST scoring procedure. This score is intended to provide an estimate of work rate during the test by incorporating both body mass and the number of touches completed within the 15-s testing period. Anthropometric and body composition variables were also examined in relation to test performance.

### 2.5. Statistical Analysis

Descriptive statistics were calculated for all variables. Differences between the standard and modified hand positions were examined for repetitions, score, and power. Test–retest reliability for each condition was assessed using the intraclass correlation coefficient (ICC), together with the standard error of measurement (SEM) and minimal detectable change (MDC) [16] (Weir, 2005). The MDC at the 95% confidence level was calculated as: MDC95 = SEM × 1.96 × √2 [17]. Associations between test performance and anthropometric/body composition variables were assessed using Pearson’s correlation coefficient [18]. Variables showing significant associations were entered into regression analyses to identify predictors of performance in each condition. Statistical significance was set at *p* < 0.05. All statistical analyses were performed using IBM SPSS Statistics for Windows, version 29.0 (IBM Corp., Armonk, NY, USA).

## 3. Results

### 3.1. Participant Characteristics

Sixty-five healthy adults participated in the study, including 44 males and 21 females. Descriptive characteristics of the sample are presented in Table 1.

### 3.2. Comparison Between Standard and Modified Positions

Performance in the modified hand position was significantly better than in the standard position across all primary outcomes. Participants achieved a greater mean number of touches in the modified condition compared with the standard condition. Similarly, both the standard score and power score were significantly higher in the modified position. These findings indicate that reducing hand distance to 50% of arm span improved CKCUEST performance. However, the effect sizes were small to moderate, indicating that the practical magnitude of the improvement was meaningful but not large. Detailed results for all three trials, as well as average values, standard score, and power, are presented in Table 2.

### 3.3. Reliability of the Two Conditions

Both testing conditions demonstrated excellent reliability. The standard condition yielded an ICC of 0.944 (95% CI: 0.921–0.961), whereas the modified condition yielded an ICC of 0.946 (95% CI: 0.924–0.963). The SEM and MDC values were slightly lower in the modified condition, suggesting marginally better measurement stability and sensitivity to change compared with the standard condition. Full reliability data are presented in Table 3.

### 3.4. Protocol-Dependent Group Comparisons

Because male and female participants performed the CKCUEST using different body positions, these analyses should not be interpreted as direct physiological sex comparisons. Instead, they are reported as protocol-dependent group comparisons. In the standard condition, the female group completed fewer touches than the male group across all trials and in the average number of touches. The female group also demonstrated lower standard scores and lower power values. In the modified condition, group differences in the number of touches and standard score were no longer statistically significant, although power remained significantly lower in the female group. These findings suggest that the individualized hand position reduced protocol-dependent group differences in repetitions and standard score, but not in power. Detailed sex-specific comparisons are presented in Table 4.

### 3.5. Correlations and Regression Analyses

Significant associations were observed between CKCUEST performance and several anthropometric and body composition variables. In both hand positions, a higher body fat percentage and greater visceral fat were associated with lower performance. In the standard condition, average touches were additionally associated with greater arm span, body weight, and lean body mass, whereas in the modified condition fewer associations with body size variables were observed. Similar patterns were found for score and power outcomes. Correlation coefficients are presented in Table 5. Overall, the strength and number of significant associations between anthropometric variables and CKCUEST performance were lower in the modified condition than in the standard condition. This finding provides empirical support for the interpretation that individualizing hand distance may reduce the influence of body dimensions on test performance. Given the sample size and the number of variables examined, the regression analyses should be interpreted as exploratory. These models should therefore be viewed as hypothesis-generating rather than definitive predictive models.

Regression analyses showed that body composition variables were significant predictors of performance across the different outcomes. For the average number of touches in the standard condition, body fat percentage, visceral fat, and fat mass were significant predictors, explaining 56.4% of the variance. In the modified condition, body fat percentage and visceral fat explained 42.2% of the variance. For standard score, body fat percentage and visceral fat were significant predictors in the standard condition, whereas visceral fat alone predicted performance in the modified condition. Power was best predicted by height, BMI, and body fat percentage in both conditions, with lean body mass additionally contributing in the modified condition. The regression models explained 38.4% to 70.4% of the variance in the performance outcomes. Detailed model coefficients are shown in Table 6.

## 4. Discussion

The present study investigated whether adjusting hand distance to 50% of arm span affects CKCUEST performance in healthy young adults. The main finding was that the modified position resulted in significantly better performance than the standard 36-inch position across all primary outcomes, including mean touches, standard score, and power. In addition, both protocols demonstrated excellent test–retest reliability, while the modified condition showed slightly lower SEM and MDC values. Collectively, these findings indicate that an arm-span-adjusted hand position may preserve the measurement stability of the CKCUEST while reducing some of the performance constraints associated with a fixed hand distance.

Although the differences between the standard and modified positions were statistically significant, the corresponding effect sizes were small to moderate. Therefore, the clinical or practical relevance of the modified protocol should be interpreted in terms of both statistical significance and effect magnitude. The observed improvement suggests a measurable benefit of individualized hand placement, but not a large performance change. Therefore, although the modified protocol produced statistically significant improvements, these changes should be interpreted as modest practical improvements rather than large clinical effects.

One plausible explanation for the superior performance observed in the modified condition is that reducing hand distance may lower the relative mechanical demand of the task, particularly for participants with smaller upper-extremity dimensions. However, this mechanism was not directly tested in the present study and should therefore be interpreted as a possible explanation rather than a confirmed biomechanical mechanism. In the original CKCUEST, all individuals perform the test with the hands placed 36 inches apart, regardless of height, shoulder width, or arm length. Previous work has questioned the appropriateness of this fixed setup for all body types and has suggested that narrower or shorter individuals may be placed at a disadvantage when the standard protocol is used. In particular, individualized or modified start positions have been proposed to normalize task demands and improve comparability across participants [15]. The present findings support this rationale, as performance improved in the modified condition and the associations between test outcomes and anthropometric variables became weaker compared with the standard condition. This pattern suggests that scaling the starting position to individual dimensions may reduce anthropometric bias and provide a more equitable estimate of closed-chain upper-extremity performance [15].

The protocol-dependent group comparisons provide additional context for interpreting the effect of hand position. In the standard condition, males performed better than females in repetitions and standard score, whereas these differences were no longer statistically significant in the modified condition. However, these findings should be interpreted cautiously because males and females performed the CKCUEST using different body positions, in accordance with the original test procedure. Therefore, the observed differences cannot be attributed solely to sex-related physiological factors. They may also reflect differences in task mechanics between the standard and modified push-up positions. The persistence of higher power values in males is not unexpected, since the CKCUEST power score incorporates body mass in its calculation and is therefore inherently sensitive to body-size differences [8]. Thus, while the modified protocol appears to attenuate sex-related differences in touches and normalized performance, the power variable remains partly influenced by body mass characteristics.

Another important finding was that both protocols showed excellent reliability. This is consistent with previous studies reporting high reliability for the standard CKCUEST in healthy and clinical populations, as well as for modified versions of the test [8,9,18,19]. The ICC values observed in the present study were slightly higher than, or comparable to, those reported in adolescents, asymptomatic adults, and athletes, supporting the reproducibility of both procedures. From a clinical standpoint, the slightly lower SEM and MDC values in the modified condition may indicate marginally better absolute reliability, although this difference was small. Nevertheless, the findings suggest that clinicians and researchers can use the arm-span-adjusted version without sacrificing measurement consistency. This is particularly relevant when the CKCUEST is used longitudinally to monitor rehabilitation progress, evaluate training adaptations, or support return-to-sport decision-making [9,18].

The present results also highlight the importance of body composition in CKCUEST performance. Higher body fat percentage and greater visceral fat were consistently associated with poorer performance in both conditions, and the regression models showed that body-composition variables explained a substantial proportion of the variance in repetitions, score, and power. These findings suggest that CKCUEST performance should not be interpreted solely as a measure of shoulder stability or upper-extremity neuromuscular control. Rather, the test appears to reflect a composite task requiring repeated support and transfer of body mass in a closed kinetic chain position. This interpretation is in line with biomechanical evidence showing that the CKCUEST performed in a push-up position requires alternating single-limb support, and places meaningful kinetic and kinematic demands on the upper quarter [4]. Therefore, individuals with less favorable body-composition profiles may be disadvantaged not only because of reduced relative strength or endurance, but also because of the greater demand of repeatedly moving and stabilizing a larger effective load.

The weaker relationships between anthropometric characteristics and performance in the modified condition are clinically relevant. In the standard position, greater arm span, body mass, lean body mass, and height showed stronger associations with performance, whereas these relationships were reduced after hand distance was individualized. This suggests that the modified setup may better isolate functional performance by reducing the confounding influence of body size. Such an effect is particularly desirable when comparing individuals of different sex or morphology, or when attempting to establish more representative reference values. In practical terms, the modified protocol may be especially useful in heterogeneous samples, where the standard 36-inch setup may overestimate the performance of larger individuals and underestimate that of smaller individuals [15].

Comparison with previous literature should be made cautiously because CKCUEST outcomes are influenced by participant characteristics, athletic level, hand position, and scoring procedures. Studies in athletic populations, especially collegiate overhead athletes, have often reported high performance values, likely reflecting sport-specific training and upper-extremity conditioning [14,20]. The current sample consisted of healthy young adults rather than highly trained athletes, which may explain why performance values were generally lower than those reported in some sport-specific cohorts, while still remaining within the broader range previously described for healthy individuals. Accordingly, the present findings are more likely to be applicable to young adult non-athletic or recreational populations than to elite athletes.

Overall, the present findings suggest that an arm-span-adjusted CKCUEST protocol may help reduce the influence of anthropometric characteristics on test performance and may improve comparability across individuals. However, the present study does not definitively prove that the modified protocol is fairer in all contexts. Rather, the observed improvement in performance and the weaker associations with several anthropometric variables provide preliminary support for this interpretation. The modified position improved performance, reduced sex-related differences in repetitions and standard score, and demonstrated reliability comparable to the standard protocol. These findings do not necessarily indicate that the modified version should replace the traditional 36-inch protocol in all settings, especially where direct comparison with existing normative datasets is required. However, they do suggest that in samples with substantial variation in body size, an individualized hand position may provide a fairer and potentially more clinically meaningful estimate of closed-chain upper-extremity functional performance [15].

Several limitations of the present study should be acknowledged. First, the sample was not balanced by sex, with males substantially outnumbering females. This imbalance may have reduced the precision of the group comparisons and limits the strength of any conclusions related to sex or sex-by-protocol effects. In addition, because males and females performed the test using different body positions, these comparisons should be interpreted as protocol-dependent rather than as direct evidence of physiological sex differences. Second, the study included only healthy young adults aged 18 to 33 years. Therefore, the findings cannot be generalized to adolescents, older adults, symptomatic individuals, or athletic populations with different training backgrounds, in whom CKCUEST performance and reliability may differ. Previous literature has shown that CKCUEST outcomes vary according to age, activity level, symptoms, and population characteristics, highlighting the need for caution when extrapolating these findings beyond the present sample [8,18]. Third, although the modified protocol appeared to reduce the influence of anthropometric characteristics on performance, the study did not include direct biomechanical or electromyographic assessment. As a result, the proposed explanation that the modified condition reduced mechanical demands remains inferential rather than directly tested. Future studies should combine functional performance testing with biomechanical analysis in order to clarify how individualized hand spacing affects loading patterns, force transfer, trunk control, and upper-extremity muscle demands during the CKCUEST [4]. Fourth, body composition was assessed using a field-based device rather than a laboratory reference method. Although this approach is practical and appropriate for clinical and applied settings, some estimation error is possible, particularly for variables such as visceral fat and lean body mass. Therefore, the regression models should be interpreted as clinically informative rather than definitive physiological models. Finally, because the study used a cross-sectional design, it cannot determine whether the modified protocol is more sensitive to change over time, more predictive of shoulder injury, or more useful for return-to-sport decisions than the standard protocol. These questions require prospective and longitudinal investigation [9]. An additional limitation of the present study is the relatively small sample size. Although the number of participants is comparable to that of similar studies in the literature, it may limit the statistical power of some analyses and restrict the generalizability of the findings. Therefore, the results should be considered preliminary and interpreted with caution. In addition, the regression models included several anthropometric and body-composition variables in a relatively small sample. Some predictors, such as BMI, body fat percentage, fat mass, and lean body mass, are conceptually and statistically related. Therefore, multicollinearity and model instability cannot be excluded. The regression findings should be interpreted as exploratory and require confirmation in larger samples. Furthermore, because several predictors were included in the regression models relative to the sample size, the possibility of overfitting cannot be excluded. Therefore, the relatively high proportions of explained variance should be interpreted with caution.

## 5. Conclusions

The findings of the present study provide clear answers to the research questions and support the proposed hypotheses. Specifically, the results confirmed that an arm-span-adjusted hand position significantly improves CKCUEST performance compared with the standard 36-inch position (H1 supported). In addition, protocol-dependent group differences between male and female participants in repetitions and standard score were reduced in the modified condition, supporting the second hypothesis (H2). However, because males and females performed the test using different body positions, these findings should not be interpreted as direct evidence of reduced physiological sex differences. Furthermore, anthropometric and body composition variables were significantly associated with performance outcomes, with weaker associations observed in the modified condition, partially supporting the third hypothesis (H3).

These findings indicate that adjusting hand position to individual arm span may reduce the influence of anthropometric characteristics and may provide a more comparable assessment across individuals with different body dimensions. From a practical perspective, the modified protocol may be preferable when the goal is to improve fairness of assessment across individuals with different body dimensions, while the standard protocol may still be appropriate when comparison with previously published normative data is required. However, given the relatively small sample size, the findings should be considered preliminary and interpreted with caution.

Future research should establish population-specific reference values for the arm-span-adjusted version and further examine its clinical utility in rehabilitation monitoring, injury risk screening, and return-to-sport decision-making. Future studies should also include larger and more diverse samples, with balanced representation across sex and different activity levels, and should consider these factors at the research design stage to enhance the generalizability of the findings.

## Figures and Tables

**Table 1:** Descriptive characteristics of the sample. Values are presented as mean ± SD for continuous variables and *n* (%) for categorical variables.

Variable	Total (*n* = 65)	Male (*n* = 44)	Female (*n* = 21)
Age (years)	24.2 ± 3.9	23.8 ± 3.4	25.0 ± 4.9
Height (cm)	172.1 ± 9.7	176.5 ± 7.9	162.8 ± 5.9
Weight (kg)	73.7 ± 13.1	79.6 ± 10.9	61.2 ± 7.1
BMI (kg/m^2^)	24.8 ± 3.3	25.6 ± 3.3	23.2 ± 2.6
Arm length (cm)	176.0 ± 11.4	181.7 ± 8.3	164.0 ± 6.9
Body fat (%)	22.8 ± 8.3	20.0 ± 7.8	28.5 ± 6.6
Fat mass (kg)	17.5 ± 8.0	16.6 ± 8.4	19.4 ± 7.0
Muscle mass (kg)	55.3 ± 10.7	61.4 ± 7.3	42.6 ± 4.1
Non-fat body weight (%)	77.2 ± 8.3	80.0 ± 7.8	71.5 ± 6.6
Visceral fat (level)	7.8 ± 4.5	9.5 ± 4.2	4.2 ± 2.8
Dominant arm, *n* (%)			
Right	50 (76.9%)	32 (72.7%)	18 (85.7%)
Left	15 (23.1%)	12 (27.3%)	3 (14.3%)

Abbreviations: BMI, body mass index; SD, standard deviation.

**Table 2:** Effect of hand position on repetitions per trial, mean number of repetitions, standard score, and power.

	Standard Position	Modified Position	*p*-Value	Mean Difference (SEM)	Effect Size (Cohen’s d, 95% CI)
Trial 1	23.2 (4.85)	24.2 (4.71)	0.019	−1.0 (0.42)	−0.30 (−0.547, −0.05)
Trial 2	22.9 (4.77)	24.8 (4.70)	0.001	−1.9 (0.40)	−0.60 (−0.859, −0.331)
Trial 3	22.8 (5.00)	24.3 (4.71)	0.003	−1.5 (0.48)	−0.39 (−0.638, −0.133)
Average	23.0 (4.62)	24.4 (4.47)	0.001	−1.5 (0.35)	−0.52 (−0.78, −0.262)
Standard score	0.3 (0.06)	0.4 (0.07)	0.001	−0.1 (0.01)	−0.55 (−0.804, −0.283)
Power	77.1 (22.00)	81.1 (18.29)	0.001	−4.0 (1.10)	−0.45 (−0.703, −0.192)

Abbreviations: SEM, standard error of measurement; CI, confidence interval; *p*-value, probability value.

**Table 3:** Reliability coefficients for standard and modified execution of the test.

	ICC	95% CI	SEM	MDC	MDC as a % of Mean
Standard Position	0.944	0.921–0.961	1.895	5.25	23%
Modified Position	0.946	0.924–0.963	1.794	4.97	20%

Abbreviations: ICC, intraclass correlation coefficient; CI, confidence interval; SEM, standard error of measurement; MDC, minimal detectable change.

**Table 4:** Effect of gender on the mean, score and power in each of the two positions of the test.

	Group	Mean	SD	Mean Difference	SE Difference	*p*-Value
Trial 1 Standard	Female	20.381	3.993	−4.096	1.188	0.001
	Male	24.477	4.688			
Trial 2 Standard	Female	20.048	4.341	−4.225	1.158	0.001
	Male	24.273	4.379			
Trial 3 Standard	Female	20.048	5.249	−4.043	1.235	0.002
	Male	24.091	4.355			
Trial 1 Modified	Female	22.857	4.586	−1.938	1.234	0.121
	Male	24.795	4.683			
Trial 2 Modified	Female	24.333	5.102	−0.735	1.254	0.56
	Male	25.068	4.541			
Trial 3 Modified	Female	23.952	4.577	−0.502	1.256	0.691
	Male	24.455	4.81			
Avg Standard	Female	20.159	4.2	−4.122	1.12	0.001
	Male	24.28	4.233			
Avg Modified	Female	23.714	4.57	−1.058	1.188	0.376
	Male	24.773	4.435			
Score Standard	Female	0.314	0.059	−0.037	0.017	0.031
	Male	0.35	0.064			
Score Modified	Female	0.37	0.069	0.012	0.019	0.535
	Male	0.358	0.072			
Power Standard	Female	56.132	14.032	−30.965	4.4	0.001
	Male	87.097	17.651			
Power Modified	Female	65.655	13.764	−22.761	3.961	0.001
	Male	88.416	15.446			

Abbreviations: SD, standard deviation; SE, standard error; *p*-value, probability value; Avg, average.

**Table 5:** Correlation of mean, score and power in the two positions of the test with anthropometric and body composition variables.

		Height	Weight	BMI	Fat (%)	Fat (kgr)	LBM-kgr	Visceral Fat
Avg Standard Position	Pearson’s r	0.359	≥0.05	≥0.05	−0.642	−0.515	0.412	−0.468
	*p*-value	0.003			0.001	0.001	0.001	0.001
Avg Modified Position	Pearson’s r	≥0.05	≥0.05	≥0.05	−0.481	−0.532	≥0.05	−0.614
	*p*-value				0.001	0.001		0.001
Score Standard Position	Pearson’s r	≥0.05	≥0.05	≥0.05	−0.55	−0.506	≥0.05	−0.532
	*p*-value				0.001	0.001		0.001
Score Modified Position	Pearson’s r	n/a	−0.366	≥0.05	−0.321	−0.461	≥0.05	−0.62
	*p*-value		0.003		0.009	0.001		0.001
Power Standard position	Pearson’s r	0.63	n/a	0.441	−0.464	≥0.05	0.81	≥0.05
	*p*-value	0.001		0.001	0.001		0.001	
Power Modified Position	Pearson’s r	0.444	n/a	0.438	−0.426	≥0.05	0.667	≥0.05
	*p*-value	0.001		0.001	0.001		0.001	

Abbreviations: BMI, body mass index; Fat (%), body fat percentage; Fat (kgr), fat mass in kilograms; LBM-kgr, lean body mass in kilograms; Avg, average; Pearson’s r, Pearson correlation coefficient; *p*-value, probability value; n/a, not applicable. Exact *p*-values are reported for significant associations, while non-significant results are presented as *p* ≥ 0.05 for clarity.

**Table 6:** Linear regression models of performance variables with anthropometric and body composition variables.

Outcome	R	R^2^	Predictor	B	SE	t	*p*	Model *p*
Avg Touches Standard Position	0.753	0.564	Intercept	30.468	1.047	29.095	<0.001	0.001
			Fat (%)	−0.704	0.112	−6.289	<0.001	
			Visceral fat	−1.036	0.233	−4.445	<0.001	
			Fat (kgr)	0.746	0.180	4.157	<0.001	
Avg Touches Modified Position	0.649	0.422	Intercept	30.458	1.162	26.214	<0.001	0.001
			Fat (%)	−0.125	0.057	−2.182	0.033	
			Visceral fat	−0.831	0.184	−4.514	<0.001	
Score Standard Position	0.628	0.395	Intercept	0.433	0.017	25.360	<0.001	0.001
			Fat (%)	−0.003	0.001	−3.386	0.001	
			Visceral fat	−0.008	0.003	−3.078	0.003	
Score Modified Position	0.620	0.384	Intercept	0.428	0.013	33.996	<0.001	0.001
			Visceral fat	−0.016	0.003	−6.264	<0.001	
Power Standard Position	0.839	0.704	Intercept	−139.040	33.285	−4.177	<0.001	0.001
			Height	0.858	0.185	4.640	<0.001	
			BMI	3.778	0.504	7.498	<0.001	
			Fat (%)	−1.186	0.220	−5.393	<0.001	
Power Modified Position	0.794	0.631	Intercept	−301.120	88.531	−3.401	0.001	0.001
			Height	2.148	0.588	3.656	<0.001	
			BMI	9.417	1.896	4.967	<0.001	
			Fat (%)	−2.851	0.551	−5.172	<0.001	
			LBM-Kgr	−2.873	0.867	−3.312	0.002	

Abbreviations: R, multiple correlation coefficient; R^2^, coefficient of determination; SE, standard error; t, t statistic; *p*, probability value; Avg, average; Fat (%), body fat percentage; Fat (Kgr), fat mass in kilograms; LBM-Kgr, lean body mass in kilograms; BMI, body mass index.

## Data Availability

The original contributions presented in this study are included in the article. Further inquiries can be directed to the corresponding author.

## Abbreviations

The following abbreviations are used in this manuscript:

CKCUEST	Closed Kinetic Chain Upper Extremity Stability Test
ICC	Intraclass Correlation Coefficient
SEM	Standard Error of Measurement
MDC	Minimal Detectable Change
CI	Confidence Interval
BMI	Body Mass Index
LBM	Lean Body Mass
SD	Standard Deviation
SE	Standard Error
R	Correlation Coefficient
R2	Coefficient of Determination
Avg	Average
Fat (%)	Body Fat Percentage
Fat (kgr)	Body Fat Mass in Kilograms
n/a	Not Applicable
ns	Not Significant
p	Probability Value
*p*-value	Probability Value
r	Pearson’s Correlation Coefficient
